# Lurasidone in Children and Adolescents with Bipolar Depression Presenting with Mixed (Subsyndromal Hypomanic) Features: *Post Hoc* Analysis of a Randomized Placebo-Controlled Trial

**DOI:** 10.1089/cap.2020.0018

**Published:** 2020-12-03

**Authors:** Manpreet K. Singh, Andrei Pikalov, Cynthia Siu, Michael Tocco, Antony Loebel

**Affiliations:** ^1^ Psychiatry and Behavioral Sciences, Stanford University, Stanford, California, USA.; ^2^ Sunovion Pharmaceuticals, Inc., Marlborough, Massachusetts, and Fort Lee, New Jersey, USA.; ^3^ COS and Associates Ltd., Central, Hong Kong, People's Republic of China.

**Keywords:** bipolar depression, mixed (subsyndromal hypomanic) features, children and adolescents, lurasidone

## Abstract

***Objectives:*** To evaluate the efficacy and safety of lurasidone in the treatment of children and adolescents with bipolar depression presenting with mixed (subsyndromal hypomanic) features.

***Methods:*** Patients, 10–17 years of age, with a Diagnostic and Statistical Manual of Mental Disorders, 5th ed. (DSM-5), diagnosis of bipolar I depression were randomized to 6 weeks of double-blind treatment with once-daily flexible doses of lurasidone 20–80 mg or placebo. The presence of mixed (subsyndromal hypomanic) features in this pediatric bipolar depression trial was defined as a Young Mania Rating Scale score of 5 or greater at study baseline. Key efficacy measures included change from baseline to week 6 in the Children's Depression Rating Scale-Revised (CDRS-R) score (primary endpoint) and Clinical Global Impressions-Bipolar Severity (CGI-BP-S) score, using a mixed model for repeated measures analysis.

***Results:*** At baseline, subsyndromal hypomanic features were present in 54.2% of patients. Treatment with lurasidone (vs. placebo) was associated with significantly greater reductions in CDRS-R scores at week 6, independent of the presence (−21.5 vs. −15.9, *p* < 0.01; effect size *d* = 0.43) or absence (−20.5 vs. −14.9, *p* < 0.01; *d* = 0.44) of subsyndromal hypomanic features. Likewise, lurasidone (vs. placebo) was associated with significantly greater reductions in CGI-BP-S scores at week 6, independent of the presence (−1.6 vs. −1.1, *p* < 0.001, *d* = 0.51) or absence (−1.3 vs. −1.0, *p* = 0.05; *d* = 0.31) of these subsyndromal hypomanic features. Rates of protocol-defined treatment-emergent hypomania or mania were similar for lurasidone and placebo in patients with (lurasidone 8.2% vs. placebo 9.0%) or without subsyndromal hypomanic features (lurasidone 1.3% vs. placebo 3.7%).

***Conclusions:*** In this *post hoc* analysis of a randomized placebo-controlled trial, lurasidone was found to be efficacious in the treatment of child and adolescent patients with bipolar depression who presented with mixed (subsyndromal hypomanic) features. No differences in safety profile, including the risk of treatment-emergent mania, were observed in patients with or without subsyndromal hypomanic features in this study.

## Introduction

Pediatric bipolar disorder is a disabling condition associated with long-term psychiatric comorbidity and physical health problems (Findling et al. 2001; Youngstrom et al. 2005; Axelson et al. 2006) that increase the risk of suicide attempt (Goldstein et al. 2005; Algorta et al. 2011), substance abuse, and impaired functioning (Goldstein et al. 2009; Keenan-Miller and Miklowitz 2011). The prevalence of bipolar disorder in children and adolescents ranges from 1% to 3% (Chang 2007; Merikangas et al. 2007; Van Meter et al. 2011; Birmaher 2013) and may be higher if subsyndromal spectrum conditions are included (Dilsaver et al. 2005; Birmaher and Axelson 2009). Further, up to 60% of adult patients with bipolar disorder experienced onset of mood symptoms before 21 years of age (Chengappa et al. 2003; Baldessarini et al. 2010).

Symptom profiles associated with mood disorder are complex and heterogeneous. In both unipolar and bipolar depression, manic symptoms often occur during episodes of depression (Zimmermann et al. 2009; Azorin et al. 2012; Swann et al. 2013; McIntyre et al. 2014; Suppes et al. 2016). In the systematic treatment enhancement program for bipolar disorder study, only one-third of the subjects were reported to have no manic symptoms during their depressive episode (Goldberg et al. 2009).

Bipolar I depression may be associated with mixed features, defined in the Diagnostic and Statistical Manual of Mental Disorders, 5th ed. (DSM-5; American Psychiatric Association 2013), as meeting full diagnostic criteria for a recent bipolar depressive episode and also experiencing at least three symptoms of mania or hypomania during the majority of the depressive episode. Compared with other subtypes of bipolar disorder, mixed features are frequently associated with worse symptoms, more frequent recurrence of acute episodes, poorer functioning and quality of life, increased risk of psychosis and suicidality, and greater challenges in finding effective treatments (Algorta et al. 2011; Frazier et al. 2017). However, in pediatric populations, mania symptoms may not meet clear diagnostic criteria due to lack of sufficient symptoms or a sufficient time frame. Nevertheless, the presence of mania symptoms during depression, however subsyndromal, can result in significant impairment in functioning and may complicate treatment selection due to concerns for treatment-emergent activation. Consequently, a systematic approach to an accurate diagnosis is critical to select effective and well-tolerated treatments, without unintended antidepressant-related activation or worsening of manic symptoms.

There has been limited research on the pharmacological management of bipolar depression with associated manic symptoms in youth or adults (Cuomo et al. 2017; ref for youth). Although there are several randomized controlled trials that support treatment for acute manic and mixed episodes in pediatric bipolar disorder (Findling 2016), there are no studies that have investigated the efficacy and safety of treating bipolar I depression with subsyndromal mania in this population. Therefore, there is a pressing need for effective evidence-based treatments for complex mood presentations in youth with bipolar depression.

Lurasidone is a second-generation antipsychotic agent that is approved for the treatment of bipolar depression in adults as monotherapy and as adjunctive therapy with lithium or valproate (Loebel et al. 2014a, b) and in children and adolescents as monotherapy (Delbello et al. 2017) in the United States and elsewhere. Lurasidone has also demonstrated efficacy for the treatment of adults with unipolar depression presenting with subsyndromal hypomanic symptoms (McIntyre et al. 2015; Suppes et al. 2016). The objective of this *post hoc* analysis was to evaluate the efficacy and safety of lurasidone in children and adolescents with mixed (subsyndromal hypomanic) features during episodes of bipolar depression.

## Methods

### Patients and study design

This *post hoc* analysis was based on a previously reported placebo-controlled, lurasidone monotherapeutic study in children and adolescents with bipolar depression (Clinicaltrials.gov identifier: NCT02046369) (Delbello et al. 2017). This multicenter study was conducted from March 2014 to October 2016 over 30 months of recruitment at 64 clinical sites in 11 countries. The study was approved by the institutional review board at each investigational site and conducted in accordance with the International Conference on Harmonization Good Clinical Practice guidelines and with the ethical principles of the Declaration of Helsinki. Informed assent and consent were obtained from all patients and their legal guardians, respectively, at study entry and before commencement of any study procedures. Pediatric patients 10–17 years of age, inclusive, with a DSM-5 diagnosis of bipolar I depression, with or without rapid cycling disease course, and without psychotic features, were randomized to 6 weeks of double-blind treatment with a once-daily flexible dose of lurasidone, 20–80 mg, or placebo (Debello et al. 2017). Eligible patients were required to have a Young Mania Rating Scale (YMRS) score of ≤15, with a YMRS item 1 (elevated mood) score ≤2 at screening and baseline. Subjects randomized to lurasidone were started on 20 mg/day during week 1 and then permitted to dose flexibly from 20 to 80 mg/day.

The eligibility screening procedure in this study required confirmation of the bipolar I disorder diagnosis verified by a trained clinician at the time of screening and by means of the Schedule for Affective Disorders and Schizophrenia for School-age Children (K-SADS-PL) structured clinical interview. The current episode of major depression associated with bipolar I disorder was confirmed and documented by the investigator. In an effort to improve the consistency of subject assessment and rater precision across sites, an independent rater qualification service, Bracket, in collaboration with the sponsor, developed a credential and experience survey to identify raters with appropriate experience and developed an educational program to train these raters for reliability. The training provided consistent rater training and standardization for the Children's Depression Rating Scale-Revised (CDRS-R), Clinical Global Impressions-Bipolar Severity of Depression Score (CGI-BP-S), YMRS, Pediatric Anxiety Rating Scale (PARS), Children's Global Assessment Scale (CGAS), and the other rating scales used in this study. In addition, raters were qualified on the CDRS-R assessment. All diagnoses and symptom assessments were conducted by qualified raters with demonstrated inter-rater reliability (DelBello et al. 2017).

### Definition of subsyndromal hypomania features

Based on a median split of the study sample, a YMRS (Young et al. 1978) score ≥5 was used to define the presence of mixed (i.e., subsyndromal hypomanic) features at study baseline. We also conducted a sensitivity analysis using an alternative definition of subsyndromal hypomanic features that required the presence of two or more YMRS symptoms, each with a severity score of ≥2 at study baseline (Goldberg et al. 2009; Zimmermann et al. 2009; Azorin et al. 2012; Swann et al. 2013; Tohen et al. 2014).

### Assessments

The primary endpoint in the underlying study was the change in the CDRS-R (Poznanski and Mokros 1996) total score compared with placebo from baseline to week 6 (Delbello et al. 2017). Key secondary endpoints included a change in Clinical Global Impressions-Bipolar Version, Severity of Illness (CGI-BP-S) score (depression) (Guy 1976) from baseline to week 6 and rates of protocol-defined treatment-emergent hypomania or mania defined *a priori* as (1) a YMRS score ≥16 at any two consecutive postbaseline visits or at the final visit or (2) an adverse event of mania or hypomania. Additional outcome assessments included the YMRS, the Pediatric Anxiety Rating Scale (PARS, 2002), the Children's Global Assessment Scale (CGAS) (Bird et al. 1987), and Pediatric Quality of Life Enjoyment and Satisfaction Questionnaire (PQ-LES-Q) (Endicott et al. 2006).

### Statistical methods

The population for the current analysis included all randomized subjects who received at least one dose of study medication and had at least one postbaseline assessment for any efficacy variable. The current *post hoc* analysis tested whether the antidepressant efficacy of lurasidone (vs. placebo) was similar in patients with or without mixed (subsyndromal hypomanic) features at the study baseline. The presence or absence of mixed (subsyndromal hypomanic) features was used to stratify the data and evaluated using a mixed model for repeated measures (MMRM), which included terms for baseline score, age strata, treatment, visit, and treatment by visit and interaction terms for mixed features, treatment, and visit. Sensitivity analysis was conducted using the analysis of covariance (ANCOVA) model, which included terms for baseline score, age strata, and treatment, a stratification factor by the presence (or absence) of subsyndromal hypomanic features at baseline, and interaction terms for subsyndromal hypomanic features and treatment. The association between improvement in CDRS-R and YMRS scores with lurasidone (vs. placebo) was explored using mediation analysis by including the change in the YMRS score in the ANCOVA model. Effect size was calculated as least squares mean (LSM) difference between the treatment groups divided by model estimate of pooled standard deviation (SD). All statistical tests were conducted using a two-tailed test at the 0.05 significance level. Treatment group means are reported as LSM ± SD.

## Results

### Baseline characteristics

**FIG. 1.**
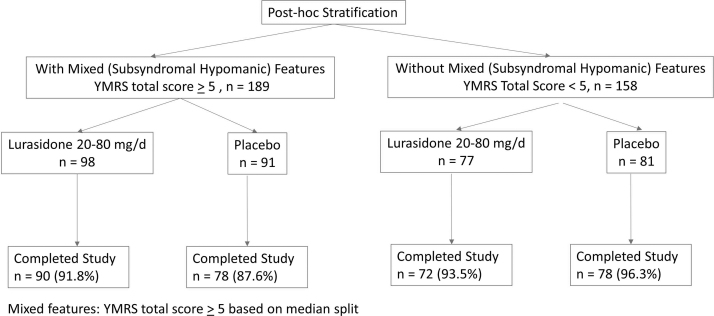
Subject disposition (safety population).

Figure 1 shows the subject disposition of the study. At study baseline, mixed (subsyndromal hypomanic) features were present in 186 (54.2%) patients (YMRS score ≥5 based on median split). The subgroup with subsyndromal hypomanic features (vs. subgroup without subsyndromal hypomanic features) was more likely to be male (58.1% vs. 42.7%), have a history of attention-deficit/hyperactivity disorder (ADHD) (30.6% vs. 15.9%), and have higher baseline YMRS scores (7.9 ± 0.2 vs. 2.2 ± 0.1) (Table 1). Other clinical and demographic features were comparable between patient subgroups with or without subsyndromal hypomanic features (as assessed by the YMRS score), including baseline depressive symptom severity, age at onset of bipolar disorder, and past number of hospitalizations for bipolar disorder. At study baseline, the number of subjects using stimulants (for the treatment of ADHD) was 26 (14.0%) in the group with mixed (subsyndromal hypomanic) features (YMRS >5) and 13 (8.3%) in the group without mixed (subsyndromal hypomanic) features.

**Table 1.** Baseline Characteristics

	With subsyndromal hypomanic features (YMRS ≥5)		Without subsyndromal hypomanic features (YMRS <5)	
	Lurasidone 20–80 mg/day	Placebo	Lurasidone 20–80 mg/day	Placebo
	n = 97	n = 89	n = 76	n = 81
Age, mean ± SD, years	13.6 ± 2.2	14.1 ± 2.2	14.8 ± 2.0	14.5 ± 1.9
Age at onset of bipolar disorder, mean ± SD, years	11.8 ± 2.7	11.6 ± 2.8	13.4 ± 2.6	12.8 ± 2.4
Male, *n* (%)	54 (55.7%)	54 (60.7%)	34 (44.7%)	33 (40.7%)
Race, *n* (%)				
White	69 (71.1%)	58 (65.2%)	65 (85.5%)	67 (82.7%)
Black/African American	14 (14.4%)	18 (20.2%)	1 (1.3%)	0
Asian	2 (2.1%)	3 (3.4%)	5 (6.6%)	1 (1.2%)
Other	12 (12.4%)	10 (11.2%)	5 (6.6%)	13 (16.1%)
One or more hospitalizations for bipolar disorder	27 (27.8%)	25 (28.1%)	17 (22.4%)	24 (29.6%)
ADHD diagnosis	30 (30.9%)	27 (30.3%)	13 (17.1%)	12 (14.8%)
ADHD treated with stimulants	13/30	13/27	5/13	8/12
CDRS-R total score, mean ± SD	59.3 ± 7.9	58.1 ± 8.6	59.2 ± 8.7	59.1 ± 7.9
CGI-BP-S depression score, mean ± SD	4.7 ± 0.7	4.5 ± 0.6	4.5 ± 0.6	4.4 ± 0.5
YMRS total score, mean ± SD	8.2 ± 2.8	7.6 ± 2.1	2.1 ± 1.5	2.3 ± 1.5

ADHD, attention-deficit/hyperactivity disorder; CDRS-R, Children's Depression Rating Scale-Revised; CGI-BP-S, Clinical Global Impressions-Bipolar Severity; YMRS, Young Mania Rating Scale; SD, standard deviation.

### Efficacy

**FIG. 2.**
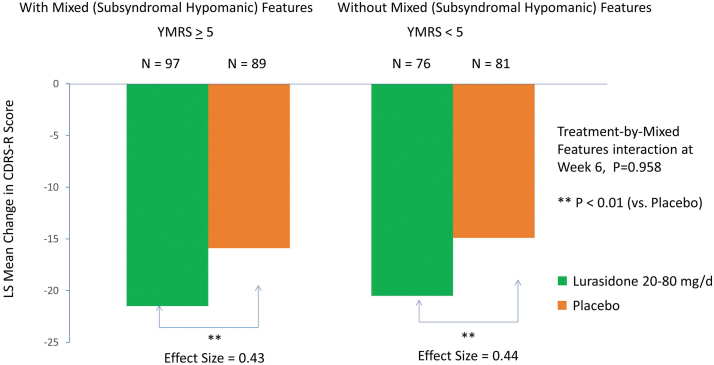
Change in Children's Depression Rating Scale-Revised in patients with and without mixed (subsyndromal hypomanic) features (based on the YMRS score). YMRS, Young Mania Rating Scale.

**FIG. 3.**
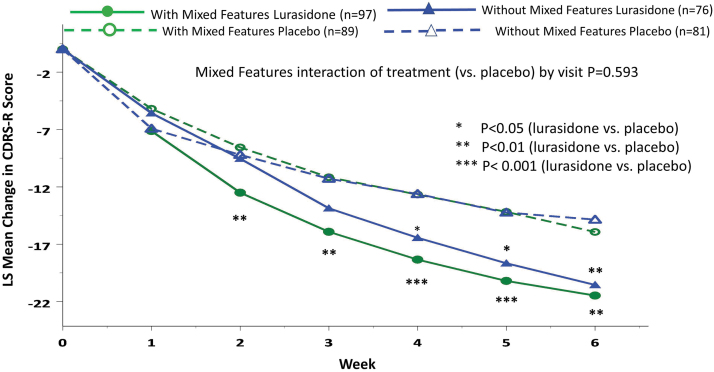
Change in Children's Depression Rating Scale-Revised trajectory in patients with and without mixed (subsyndromal hypomanic) features (based on the YMRS score). YMRS, Young Mania Rating Scale.

Treatment with lurasidone (vs placebo) was associated with significantly greater reduction in CDRS-R scores at week 6 in the group with subsyndromal hypomanic features (LSM ± SD −21.48 ± 12.97 vs. −15.94 ± 12.97; *p* = 0.004; effect size, 0.43, MMRM) and in the group without subsyndromal hypomanic features (−20.55 ± 12.83 vs. −14.86 ± 12.83; *p* = 0.006; effect size, 0.44, MMRM) (interaction between treatment and mixed features, effect at week 6: *p* = 0.958, *F* = 0.0025, df = 1, 331, MMRM; *p* = 0.768, *F* = 0.09, df = 1, 322, ANCOVA) (Fig. 2; Table 2). There was no significant interaction between lurasidone treatment and the presence (vs. absence) of subsyndromal hypomanic features by visit for change in CDRS-R scores (*p* = 0.593, *F* = 0.74, df = 5, 331, MMRM). Lurasidone was associated with significantly greater reductions in CDRS-R scores from baseline compared with placebo, starting at week 2, in patients with subsyndromal hypomanic features (Fig. 3). In patients without subsyndromal hypomanic features, lurasidone was superior to placebo in change from baseline in CDRS-R scores from week 4 onward (Fig. 3).

**Table 2.** Efficacy Outcome by the Presence or Absence of Mixed Features at Study Baseline

	With subsyndromal hypomanic features (YMRS ≥5)				Without subsyndromal hypomanic features (YMRS <5)			
	Lurasidone* n* = 97		Placebo* n* = 89		Lurasidone* n* = 76		Placebo* n* = 81	
*LS mean change at week 6*	*LS mean*	*SD*	*LS mean*	*SD*	*LS mean*	*SD*	*LS mean*	*SD*
CDRS-R total	−21.48^**^	12.96	−15.94	12.96	−20.55^**^	12.83	−14.86	12.83
CGI-BP-S depression score	−1.61^***^	1.04	−1.08	1.04	−1.33^*^	1.03	−1.01	1.03
YMRS total	−2.98^***^	3.47	−1.23	3.47	−0.98	3.42	−1.26	3.42
PARS	−2.97	4.98	−1.44	4.98	−3.85^*^	4.93	−3.06	4.93
CGAS	14.08^**^	1.28	9.82	1.33	14.02^**^	1.34	8.87	1.33
Pediatric Q-LES-Q	11.42	12.72	8.50	12.72	12.30^*^	12.50	7.39	12.50
	*Secondary analysis*							
	*With subsyndromal hypomanic features*				*Without subsyndromal hypomanic features*			
	*Baseline ≥2 on 2 or more YMRS items*				*Baseline ≥2 on <2 YMRS items*			
	*Lurasidone* n* = 89*		*Placebo* n* = 78*		*Lurasidone* n* = 84*		*Placebo* n* = 92*	
	*LS mean*	*SD*	*LS mean*	*SD*	*LS mean*	*SD*	*LS mean*	*SD*
CDRS-R total	−21.46^*^	12.96	−16.59	12.96	−20.60^**^	12.86	−14.36	12.86
CGI-BP-S depression score	−1.62^**^	1.04	−1.12	1.04	−1.35^*^	1.03	−0.99	1.03

^*^ *p* < 0.05 (lurasidone vs. placebo).

^**^ *p* < 0.01 (lurasidone vs. placebo).

^***^ *p* < 0.001 (lurasidone vs. placebo).

CDRS-R, Children's Depression Rating Scale-Revised; CGI-BP-S, Clinical Global Impressions-Bipolar Severity; YMRS, Young Mania Rating Scale; PARS, Pediatric Anxiety Rating Scale; CGAS, Children's Global Assessment Scale; Q-LES-Q, Quality of Life Enjoyment and Satisfaction Questionnaire; Pediatric Q-LES-Q is the percent of the maximum possible score; SD, model estimate of pooled standard deviation; LS, least squares.

Change in CDRS-R score was significantly associated with change in the YMRS score in the subgroup with subsyndromal hypomanic features (*r* = 0.49, *p* < 0.001), but not in the subgroup without subsyndromal hypomanic features (*r* = 0.10, *p* = 0.222) (treatment interaction with change in the YMRS score *p* = 0.031, *F* = 4.69, df = 1, 330). Among patients with subsyndromal hypomanic features, mediational analysis showed that 50.1% of the total lurasidone treatment effect size for improvement in the CDRS-R score at week 6 was due to the change in the YMRS score (lurasidone −2.98 ± 3.47 vs. placebo −1.23 ± 3.47) (*p* < 0.001, *F* = 12.89, df = 1, 332). Among patients without subsyndromal hypomanic features, change in the YMRS score at week 6 was not significant comparing lurasidone versus placebo treatment (Table 2).

**FIG. 4.**
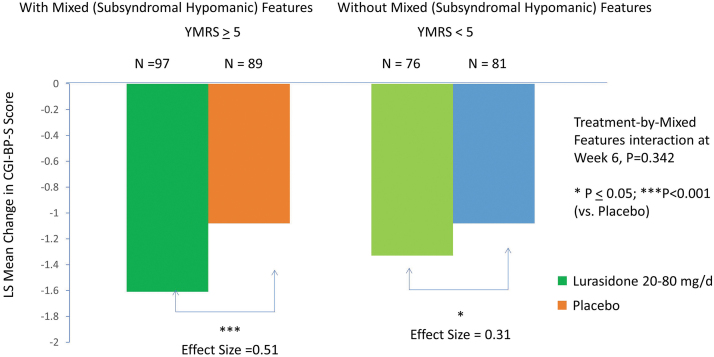
Change in Clinical Global Impression-Bipolar-Severity (CGI-BP-S) in patients with and without mixed (subsyndromal hypomanic) features (based on YMRS score). YMRS, Young Mania Rating Scale.

Lurasidone was associated with a greater effect size for reductions in CGI-BP-S scores at week 6 in the subgroup with subsyndromal hypomanic features (−1.61 vs. −1.08; *p* < 0.001; effect size 0.51) compared with the subgroup without subsyndromal hypomanic features (−1.33 vs. −1.01; *p* = 0.05; effect size 0.31) (interaction between treatment and mixed features, effect at week 6: *p* = 0.342, *F* = 0.903, df = 1, 330, MMRM; *p* = 0.281, *F* = 1.17, df = 1, 330, ANCOVA) (Fig. 4; Table 2). There was no significant interaction between treatment (vs. placebo) and the presence (vs. absence) of subsyndromal hypomanic features by visit for change in CGI-BP-S scores (*p* = 0.223, *F* = 1.40, df = 5, 330, MMRM). No statistical interaction was observed between lurasidone treatment (vs. placebo) and the presence (vs. absence) of mixed (subsyndromal hypomanic) features for PARS (interaction between treatment and mixed features, effect at week 6: *p* = 0.498, *F* = 0.462, df = 1, 321), CGAS (interaction between treatment and mixed features, effect at week 6: *p* = 0.715, *F* = 0.137, df = 1, 321), and PQ-LES-Q (*p* = 0.473, *F* = 0.518, df = 1, 320) (Table 2).

### Sensitivity analysis using alternative criteria for mixed (subsyndromal hypomanic) features

**FIG. 5.**
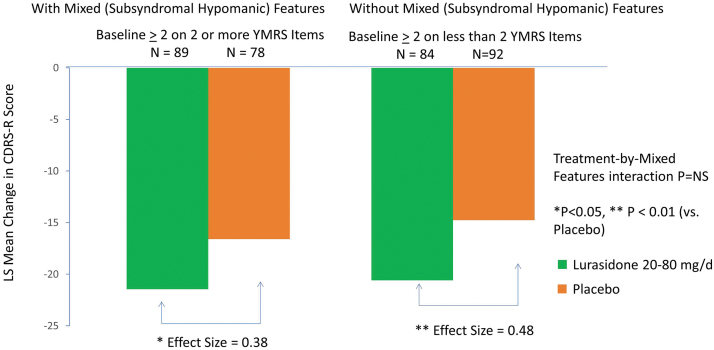
Change in Children's Depression Rating Scale-Revised (CDRS-R) in patients with and without mixed features (based on number of manic symptoms).

Consistent findings were observed using criteria for subsyndromal hypomanic features defined by a baseline severity score of ≥2 or more YMRS symptoms. There was no significant interaction between treatment (vs. placebo) and the presence (vs. absence) of subsyndromal hypomanic features by visit for change in CDRS-R (treatment-by-mixed features-by visit interaction effect *p* = 0.704, *F* = 0.59, df = 5, 330, MMRM) and CGI-BP-S (treatment-by-mixed features-by visit interaction effect *p* = 0.406, *F* = 1.02, df = 5, 330, MMRM) scores. We found that lurasidone was associated with significantly greater reductions of CDRS-R and CGI-BP-S scores from baseline to week 6 in patients with subsyndromal hypomanic features and without subsyndromal hypomanic features (≤1 YMRS symptom with a severity score of ≥2 at study baseline) (Fig. 5; Table 2).

## Safety and Tolerability

**FIG. 6.**
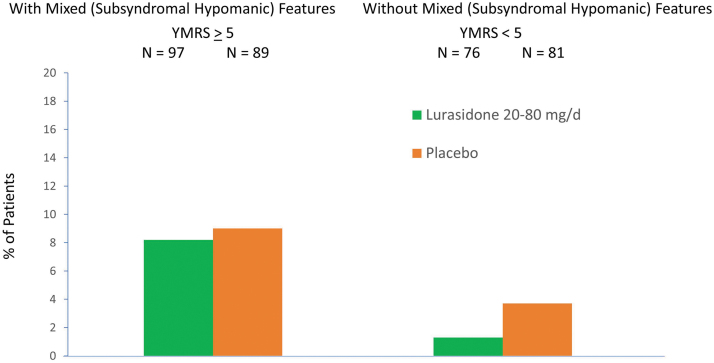
Treatment-emergent hypomania/mania rate by baseline severity of mixed features.

The rate of discontinuation was low in both the lurasidone (<9%) and placebo (<13%) groups with or without the presence of mixed (subsyndromal hypomanic) features (Fig. 1). Rates of protocol-defined treatment-emergent hypomania or mania were similar for lurasidone and placebo groups in patients with mixed (subsyndromal hypomanic) features (lurasidone 8.2% vs. placebo 9.0%) or without mixed (subsyndromal hypomanic) features (lurasidone 1.3% vs. placebo 3.7%) (Fig. 6). Table 3 shows that the frequency and proportion of treatment-emergent adverse events (TEAEs) between lurasidone and placebo were comparable in patients with and without mixed (subsyndromal hypomanic) features. Treatment-emergent suicidal ideation occurred in one patient in the placebo group without subsyndromal hypomanic features and in none of the lurasidone-treated patients (with or without subsyndromal hypomanic features).

**Table 3.** Treatment-Emergent Adverse Events with Incidence ≥5% (Safety Population)

	With subsyndromal hypomanic features (YMRS ≥5)		Without subsyndromal hypomanic features	
	Lurasidone 20–80 mg (*n* = 98)	Placebo (*n* = 91)	Lurasidone 20–80 mg (*n* = 77)	Placebo (*n* = 81)
Headache	16 (16.3%)	19 (20.9%)	9 (11.7%)	7 (8.6%)
Nausea	15 (15.3%)	5 (5.5%)	13 (16.9%)	5 (6.2%)
Vomiting	8 (8.2%)	5 (5.5%)	3 (3.9%)	1 (1.2%)
Somnolence	10 (10.2%)	5 (5.5%)	6 (7.8%)	3 (3.7%)
Weight increased	8 (8.2%)	2 (2.2%)	4 (5.2%)	1 (1.2%)
Decreased appetite	5 (5.1%)	2 (2.2%)	2 (2.6%)	2 (2.5%)
Dizziness	5 (5.1%)	4 (4.4%)	5 (6.5%)	4 (4.9%)
Fatigue	5 (5.1%)	3 (3.3%)	1 (1.3%)	1 (1.2%)
Insomnia	5 (5.1%)	3 (3.3%)	4 (5.2%)	1 (1.2%)
Influenza	0 (0%)	0 (0%)	4 (5.2%)	1 (1.2%)
Nasopharyngitis	2 (2.0%)	5 (5.5%)	5 (6.5%)	5 (6.2%)

YMRS, Young Mania Rating Scale.

## Discussion

In this *post hoc* analysis of a placebo-controlled study in children and adolescents with bipolar depression, a majority of patients (54%) met the severity criterion for mixed (i.e., subsyndromal hypomanic) features (YMRS score ≥5) at study baseline. These findings are consistent with previously reported prevalence rates of mixed features in adults with bipolar depression, which ranged from 11% to 54% depending on the study setting and criteria used (Azorin et al. 2012; Swann et al. 2013; Vieta and Valenti 2013; McIntyre et al. 2014). In this study, lurasidone treatment (vs. placebo) was effective in patients with bipolar depression presenting with or without mixed (subsyndromal hypomanic) features at the study baseline, as assessed by reduction of depressive and overall clinical symptom severity (CDRS-R and CGI-BP-S). To our knowledge, this is the first analysis of acute treatment for youth with bipolar depression with mixed (subsyndromal hypomanic) features. We found consistent results for the antidepressant efficacy of lurasidone (vs. placebo) in young patients when subsyndromal hypomanic features were defined by either the YMRS score (YMRS total score ≥5 vs. YMRS total score <5) or the presence of two or more YMRS items, each with a severity item score of ≥2. These findings suggest that therapeutic responses to lurasidone treatment were independent of the presence, number, and severity of mixed (subsyndromal hypomanic) features at study baseline, as indicated by the nonsignificant interaction effect between treatment (vs. placebo) and the presence (vs. absence) of mixed (subsyndromal hypomanic) features, utilizing two different definitions for subsyndromal hypomanic features. Results of the current *post hoc* analysis are consistent with findings reported in a previously published *post hoc* analysis of adult patients with bipolar I depression with mixed (subsyndromal hypomanic) features treated with lurasidone or placebo (McIntyre et al. 2015).

Lurasidone also showed a greater level of improvement in measures of anxiety, quality of life, and functioning, compared with placebo, in groups both with and without mixed (subsyndromal hypomanic) features. We note that lurasidone (vs. placebo) treatment was associated with improvement in both depressive and manic symptoms in this *post hoc* analysis. In the group with mixed (subsyndromal hypomanic) features, we found (based on mediational analysis) that the reduction of manic symptom severity from baseline to week 6 accounted for half of the lurasidone treatment effect size for improvement in depressive symptoms.

In the current analysis, TEAE profiles of lurasidone and placebo were comparable in groups with or without mixed (subsyndromal hypomanic) features. Rates of treatment-emergent hypomania were comparable with placebo in patients with or without mixed (subsyndromal hypomanic) features at baseline. Lurasidone was found to be generally safe and well tolerated in the parent, 6-week, double-blind placebo-controlled trial (Delbello et al. 2017) and in the subsequent 2-year, open-label follow-up study (Delbello et al. 2019).

Existing studies that examined the impact of mixed (subsyndromal hypomanic) features on treatment response in patients with bipolar depression are relatively scarce. Frye et al. (2009) suggested that even mildly elevated baseline YMRS scores in bipolar depression predispose patients to higher risks of nonresponse and treatment-emergent mania during combined antidepressant/mood stabilizer therapy. Two short-term trials of another atypical antipsychotic, olanzapine, as monotherapy or in combination with the antidepressant, fluoxetine, in the treatment of bipolar depression, reported an inverse relationship between the number and severity of mixed features and proportion of responders among enrolled patients (Tohen et al. 2014). In contrast, both the present study in children and adolescents and the previous analysis in adults (McIntyre et al. 2015) with bipolar depression associated with mixed (subsyndromal hypomanic) features found that the presence and severity of subsyndromal hypomanic features did not have a significant impact on lurasidone treatment response in patients with bipolar depression.

We note a few limitations of the current study. First, this is a *post hoc* analysis, not based on a specifically designed prospective trial to test the efficacy of lurasidone in young bipolar patients with mixed features. Second, the criteria for mixed (subsyndromal hypomanic) features used here were based on YMRS severity score. Several YMRS items (irritability, disruptive/aggressive, and distractability) are not included in the DSM-5 criteria for mixed features, but are common in pediatric bipolar presentations (Van Meter et al. 2016), especially in the context of co-occurring attention deficit with hyperactivity. Further, the DSM-5 specifier criteria mandate that mixed features be present for the majority of the depressive episode, whereas the present analysis relied on an assessment conducted at baseline to determine the presence of mixed (subsyndromal hypomanic) features. Additional studies, preferably involving prospectively designed, longer-term longitudinal trials, to further verify the impact of mixed (subsyndromal hypomanic) features on treatment of bipolar depression in different patient populations are warranted.

## Conclusions

This *post hoc* analysis of a randomized placebo-controlled trial shows that lurasidone was efficacious in children and adolescents with bipolar depression associated with mixed (subthreshold hypomanic) features. Our findings suggest that the presence of these mixed features did not influence the antidepressant efficacy of lurasidone, with significant improvement in depressive symptoms, anxiety, and quality of life found in the groups with or without subsyndromal hypomanic features. No differences in safety profile, including risk for treatment-emergent mania, were observed in patients with or without subsyndromal hypomanic features in this study. Additional prospective studies are needed to confirm these findings.

## Clinical Significance

These findings provide empirical data to support the use of lurasidone in children and adolescents with bipolar depression associated with mixed (subthreshold hypomanic) features. The presence of mixed features did not influence the antidepressant efficacy of lurasidone. No increased risk of treatment-emergent mania was found in patients with or without subsyndromal hypomanic features in this study.

## Role of the Sponsor

This study was sponsored by Sunovion Pharmaceuticals, Inc. The sponsor was involved in the design and collection of data. This publication is the work of authors. Dr. C.S. served as a statistical expert for this *post hoc* analysis.
